# Implementation Strategies for Integrating Nutrition into Mental Health Care: Insights from the Nourishing Minds Project

**DOI:** 10.1192/j.eurpsy.2026.10776

**Published:** 2026-07-31

**Authors:** R. Hiltensperger, J. Mylleri, A. Ruusunen, J. Kilian, A. S. Müller-Stierlin

**Affiliations:** 1 Institute of Epidemiology and Medical Biometry, Ulm University, Ulm, Germany; 2 Institute of Public Health and Clinical Nutrition, University of Eastern Finland, Kuopio, Finland

## Abstract

**Introduction:**

Nutrition is increasingly recognized as mental health determinant, influencing onset, course, and treatment of mental disorders through biological mechanisms (nutrients, metabolism, inflammation, gut microbiota) and psychosocial pathways. Yet structured integration of nutrition professionals and interventions into mental health care remains limited.

**Objectives:**

Nourishing Minds aimed to strengthen this integration through participatory workshops in Germany and Finland.

**Methods:**

In 2025, two workshop series were delivered in mental health clinics in Augsburg, Germany, and in Kuopio, Finland. Content was tailored to local needs. German sessions introduced foundational concepts and co-designed basic interventions. Finnish sessions addressed more advanced approaches, including integration of nutrition professionals into care pathways. Interactive methods, including World Cafés, supported reflection, experience exchange, and the development of actionable strategies.

**Results:**

In Germany, participants reported that nutrition currently plays a marginal role in mental health care despite rising patient demand. Some wards already offer small initiatives such as cooking groups. However, integration is hindered by rigid meal plans, unclear responsibilities, constraints in space and resources, and gaps in expertise. Nevertheless, interest in expanding activities was high. During the World Café, participants co-designed four concrete project ideas targeting awareness, multi-professional collaboration, and better use of existing infrastructure. Finnish workshops focused on advanced models of integrating nutrition professionals; analysis is ongoing.

**Conclusions:**

Participatory workshops proved effective in generating context-sensitive strategies and mobilizing stakeholder commitment. Findings suggest that successful integration of nutrition into mental health care requires cross-disciplinary collaboration, clear role definitions, and institutional flexibility to adapt to local resources.

**Disclosure of Interest:**

None Declared

